# Unravelling effectiveness of a nurse-led behaviour change intervention to enhance physical activity in patients at risk for cardiovascular disease in primary care: study protocol for a cluster randomised controlled trial

**DOI:** 10.1186/s13063-017-1823-9

**Published:** 2017-02-22

**Authors:** Heleen Westland, Irene D. Bos-Touwen, Jaap C. A. Trappenburg, Carin D. Schröder, Niek J. de Wit, Marieke J. Schuurmans

**Affiliations:** 1 Julius Center for Health Sciences and Primary Care, University Medical Center Utrecht, Huispost STR 6.131, PO Box 85500, Utrecht, GA 3508 The Netherlands; 2 Department of Rehabilitation, Nursing Science & Sports, University Medical Center Utrecht, Utrecht, The Netherlands

**Keywords:** Cardiovascular risk management, Behaviour change techniques, Self-management, Physical activity, Nurse-led, Behaviour change wheel, Primary care, Cluster RCT

## Abstract

**Background:**

Self-management interventions are considered effective in patients with chronic disease, but trials have shown inconsistent results, and it is unknown which patients benefit most. Adequate self-management requires behaviour change in both patients and health care providers. Therefore, the Activate intervention was developed with a focus on behaviour change in both patients and nurses. The intervention aims for change in a single self-management behaviour, namely physical activity, in primary care patients at risk for cardiovascular disease. The aim of this study is to evaluate the effectiveness of the Activate intervention.

**Methods/design:**

A two-arm cluster randomised controlled trial will be conducted to compare the Activate intervention with care as usual at 31 general practices in the Netherlands. Approximately 279 patients at risk for cardiovascular disease will participate. The Activate intervention is developed using the Behaviour Change Wheel and consists of 4 nurse-led consultations in a 3-month period, integrating 17 behaviour change techniques. The Behaviour Change Wheel was also applied to analyse what behaviour change is needed in nurses to deliver the intervention adequately. This resulted in 1-day training and coaching sessions (including 21 behaviour change techniques). The primary outcome is physical activity, measured as the number of minutes of moderate to vigorous physical activity using an accelerometer. Potential effect modifiers are age, body mass index, level of education, social support, depression, patient-provider relationship and baseline number of minutes of physical activity. Data will be collected at baseline and at 3 months and 6 months of follow-up. A process evaluation will be conducted to evaluate the training of nurses, treatment fidelity, and to identify barriers to and facilitators of implementation as well as to assess participants’ satisfaction.

**Discussion:**

To increase physical activity in patients and to support nurses in delivering the intervention, behaviour change techniques are applied to change behaviours of the patients and nurses. Evaluation of the effectiveness of the intervention, exploration of which patients benefit most, and evaluation of our theory-based training for primary care nurses will enhance understanding of what works and for whom, which is essential for further implementation of self-management in clinical practice.

**Trial registration:**

ClinicalTrials.gov identifier: NCT02725203. Registered on 25 March 2016.

**Electronic supplementary material:**

The online version of this article (doi:10.1186/s13063-017-1823-9) contains supplementary material, which is available to authorized users.

## Background

Considering the rising number of patients with one or more chronic diseases, there is an urgent need for effective interventions to enhance self-management. The aim of self-management interventions is to support patients to actively participate and take responsibility to self-manage their symptoms, treatment, physical and psychosocial consequences, behaviour and lifestyle changes in daily life [1]. Adequate self-management requires behaviour change in both patients and health care providers.

Self-management interventions have become an important part of care for patients with chronic diseases because they have been shown to positively affect health outcomes, including disease-specific outcomes, quality of life, self-management behaviour and cost-effectiveness [2–7]. However, a substantial proportion of patients does not comply with or respond to these interventions, raising new questions regarding for whom these interventions work best [8]. Trials have included different groups of patients with varying characteristics, which may also contribute to this heterogeneity in effect size. Self-management interventions might be more or less effective in specific subgroups of patients. Patients characterized as having, for example, low self-efficacy, low health-related quality of life, young age, no depressive symptoms, low education level, low income and low baseline self-management capacity tend to benefit more from self-management interventions [9–11]. However, the current evidence is inconclusive and needs further research [12].

Furthermore, heterogeneity in trial designs, intervention components, follow-up time, outcome measures, and scarcely measured and reported fidelity to study protocols may contribute to the heterogeneity in effectiveness of self-management [2, 3, 6, 13]. Therefore, further research is essential to unravel the effectiveness of self-management interventions and to explore for whom these interventions work best and whether they can be delivered as intended. For this purpose, we designed the Activate intervention, in which we focus on a large heterogeneous subgroup of patients monitored in primary care, namely patients at risk for cardiovascular disease (CVD). Patients who are at risk for CVD have at least one of the following major risk factors: high blood pressure, high cholesterol, diabetes mellitus type 2 (DM2) or a positive family history of CVD [14]. Guidelines on CVD prevention recommend pharmacotherapy and increasing the patient’s level of physical activity, healthy diet, reduction of alcohol consumption and cessation of smoking [15, 16]. The Activate intervention is targeted at increasing physical activity, which is considered to be one of the most relevant self-management components for patients at risk for CVD. Adequate physical activity is associated with a lower risk of developing diabetes and with decreased mortality, blood pressure, obesity, cholesterol level and CVD-related symptoms [17–23]. Patients are recommended to engage in at least 30 minutes of moderate activity per day for at least 5 days per week [24]. Yet, patients often fail to achieve this threshold, which emphasizes the need to change their inactive behaviour [25, 26].

Achieving behaviour change is complex and requires skills and competencies of both patients and health care professionals. In routine consultations, behaviour change support is often brief and fragmented and rarely includes recommendations on how to achieve behaviour change [27–29]. This underlines that health care providers also need to change their behaviour in order to adequately support patients in behaviour change. Nurses need to change their consultation style from traditional patient education to teaching patients problem-solving skills and supporting them in changing their behaviour, goal setting and action planning [30–32]. Unfortunately, training of health care providers does not always lead to sufficient improvement of their skills and competencies or to maintenance of the acquired skills in their daily routines [28]. Insufficient adoption of trained skills and competencies might influence the ability to adhere to study protocols and dilute the effect of the intervention [33–35].

A promising approach for developing interventions to enhance behaviour change in patients and health care providers is the comprehensive Behaviour Change Wheel (BCW) [36, 37]. The BCW incorporates 19 theoretical behaviour change frameworks. The approach begins at the hub of the wheel, where the capacity, opportunity or motivation influencing behaviour (COM-B) model is used to conduct a behavioural analysis to understand the target behaviour. The COM-B model consists of three components, capability, opportunity and motivation, which interact to generate behaviour. Surrounding the COM-B model is a layer of intervention functions to choose from that can be used to address deficits in one or more of capability, opportunity or motivation. These intervention functions can then be linked to appropriate behaviour change techniques (BCTs) [36, 37]. BCTs are regarded as active components of behaviour change and were recently defined in the Behaviour Change Technique Taxonomy v1 (BCTTv1) by Michie et al. [38]. Finally, the outer layer identifies types of policy that one can use to deliver the intervention functions [36, 37]. The Activate intervention is being developed using the BCW. Specifying the BCTs is intended to unravel the effectiveness of the intervention and to explore which patients benefit most.

The primary objective of this study is to evaluate the effect of the Activate intervention on increasing physical activity in primary care patients at risk for CVD. Secondary objectives are to

1. Evaluate the effect of the Activate intervention on sedentary behaviour, self-efficacy, level of activation and health status in primary care patients at risk for CVD
2. Identify which patient-related characteristics modify change in physical activity levels
3. Evaluate the training of nurses, treatment fidelity perceived barriers to and facilitators of implementation, and satisfaction with the Activate intervention

## Methods/design

### Design

**Fig. 1:**
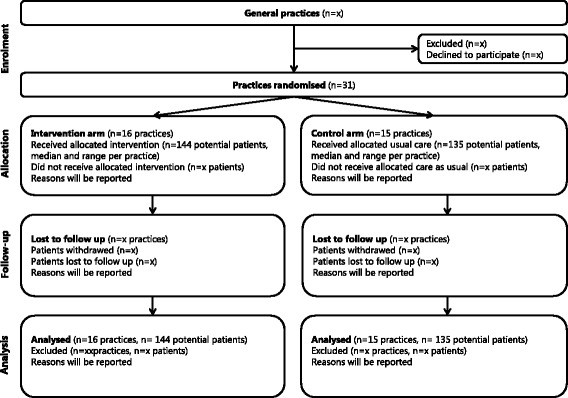
Consolidated Standards of Reporting Trials (CONSORT) diagram [65] for the Activate intervention showing participant flow through each stage of the randomised trial

We designed a two-arm cluster randomised controlled trial with the general practice as the unit of randomisation to compare the Activate intervention with care as usual. Figure 1 shows a schematic overview of the trial design.

**Fig. 2:**
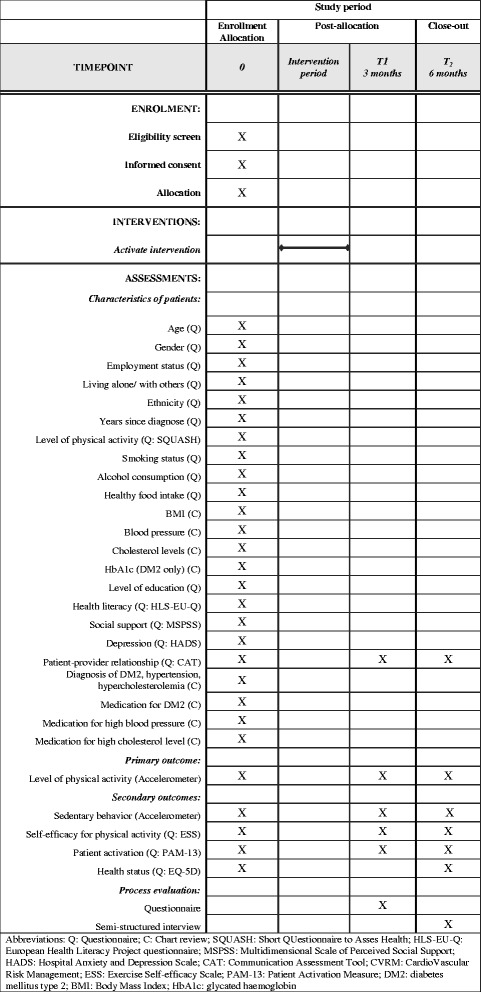
Standard Protocol Items: Recommendations for Interventional Trials (SPIRIT) figure. *Abbreviations: Q* Questionnaire, *C* Chart review, *SQUASH* Short Questionnaire to Assess Health, *HLS-EU-Q* European Health Literacy Survey Questionnaire, *MSPSS* Multidimensional Scale of Perceived Social Support, *HADS* Hospital Anxiety and Depression Scale, *CAT* Communication Assessment Tool, *CVRM* Cardiovascular risk management, *ESS* Exercise Self-efficacy Scale, *PAM-13* Patient Activation Measure short form, *DM2* diabetes mellitus type 2, *BMI* Body mass index, *HbA1c* Glycated haemoglobin

To optimize reporting of this trial and to enhance validity, this study is reported according to the 2013 Standard Protocol Items: Recommendations for Interventional Trial (SPIRIT). A SPIRIT checklist (Additional file 1) and a SPIRIT figure (Fig. 2) are provided. Protocol modifications will be reported to the institutional review board of the University Medical Center Utrecht and will be uploaded to the ClinicalTrials.gov database. The final report will be written according to the Consolidated Standards of Reporting Trials (CONSORT) extension to cluster trials.

### Participants

Patients will be recruited from general practices by primary care nurses in agreement with the general practitioner. The study population consists of adult patients at risk for CVD who are supported by a primary care nurse working in a general practice.

#### Inclusion criteria

Eligible patients will have at least one of the following risk factors as described in the Dutch guideline for cardiovascular risk management (CVRM) [16]:

- Aged 40–75 years

AND will have at least one of the following criteria:

- High blood pressure (≥140 mmHg) or already treated for high blood pressure
- High total cholesterol (≥6.5 mmol/L) or already treated for high cholesterol
- DM2
- A positive family history of CVD

AND do not meet the Dutch Norm for Healthy Exercise [24] according to the Short Questionnaire to Assess Health (SQUASH) [39].

#### Exclusion criteria

Patients are excluded from the study if they are unable to give informed consent (e.g., owing to cognitive impairment); are unable to speak, write and read Dutch; have contraindications to increasing their physical activity level (e.g., unstable angina pectoris, unstable heart failure, acute illness); or have a terminal illness or have a severe psychiatric illness or chronic disorder(s) that seriously influence their ability to improve their psychical activity level. Moreover, patients should not have participated in a structured programme conducted in a medical setting to increase their level of physical activity in the past 2 years, because including these patients might bias the effect of the Activate intervention by other prior interventions targeted at enhancing physical activity.

### Study procedures

#### Selection and recruitment

Recruitment will start with primary care nurses working in general practices located in the Netherlands. Nurses will be recruited by an invitational e-mail, by telephone and by personal contact with primary care nurses, general practitioners and practice managers until 31general practices are enrolled. Each general practitioner identifies as many patients who fulfil the inclusion and exclusion criteria determined in scheduled consultations with the nurse as needed to recruit nine or ten patients. In this way, the general practitioner guarantees a random selection of eligible patients without further selection by preference of the general practitioner or the nurse. The attending nurse will send eligible patients an envelope by mail containing an invitational letter signed and dated by their attending nurse and general practitioner, along with study information, an informed consent form (see Additional file 2) and a short self-assessment of the patient’s physical activity level using the SQUASH [39]. Patients are asked to bring the letter, informed consent form, and completed SQUASH to their next scheduled visit with the nurse. During the consultation, the nurse will check whether patients are eligible according to their level of physical activity and are willing to enrol in the study. Patients’ enrolment in the study is voluntary, and their decision about enrolment does not have any consequences for their treatment. If patients are eligible and are willing to enrol, their written informed consent will be obtained.

#### Ethical considerations

The Activate trial is ethically approved by the institutional review board of the University Medical Center Utrecht with protocol ID NL54286.041.15. Personal data will be coded and handled confidentially.

#### Informed consent

An informed consent to the postponed information procedure is being used [40], keeping patients unaware of the Activate intervention as the major study aim, randomisation and allocation of their general practice until the end of the follow-up period. With this procedure, a valid assessment of subjective outcomes can be obtained even when patients cannot be blinded to the intervention [40]. Using the modified informed consent procedure in our trial, selection bias by attrition or dropout can be reduced. A patient’s preference for allocation to the treatment arm above care as usual might result in increased dropout in the control group owing to dissatisfaction or lack of interest shown by the patient [41].

#### Randomisation and blinding

Participating general practices will be randomly allocated to the intervention group or control group after formalisation of participation. Randomisation at the level of the general practice allows evaluation of the intervention without contamination bias arising from diffusion of the intervention towards control patients. For comparability of patients’ characteristics such as employment status, literacy level, ethnicity and education, minimisation will be used to balance urbanisation areas of the general practices.

To safeguard allocation concealment, the randomisation procedure is supervised by an independent data manager and performed using web-based randomisation software. Blinding the general practices and their nurses is not possible, because nurses will perform the intervention. The investigators will be aware of the allocation as they directly communicate with the general practices and nurses about the study and are involved with the training of nurses. All patients will be informed about the assessment of their level of physical activity. Patients will be blinded as a result of the postponed information procedure. Patients who are allocated to the intervention group will only be informed about the intervention. Patients allocated to the control group will only be informed about the data collection in the control group.

### Intervention development

**Fig. 3:**
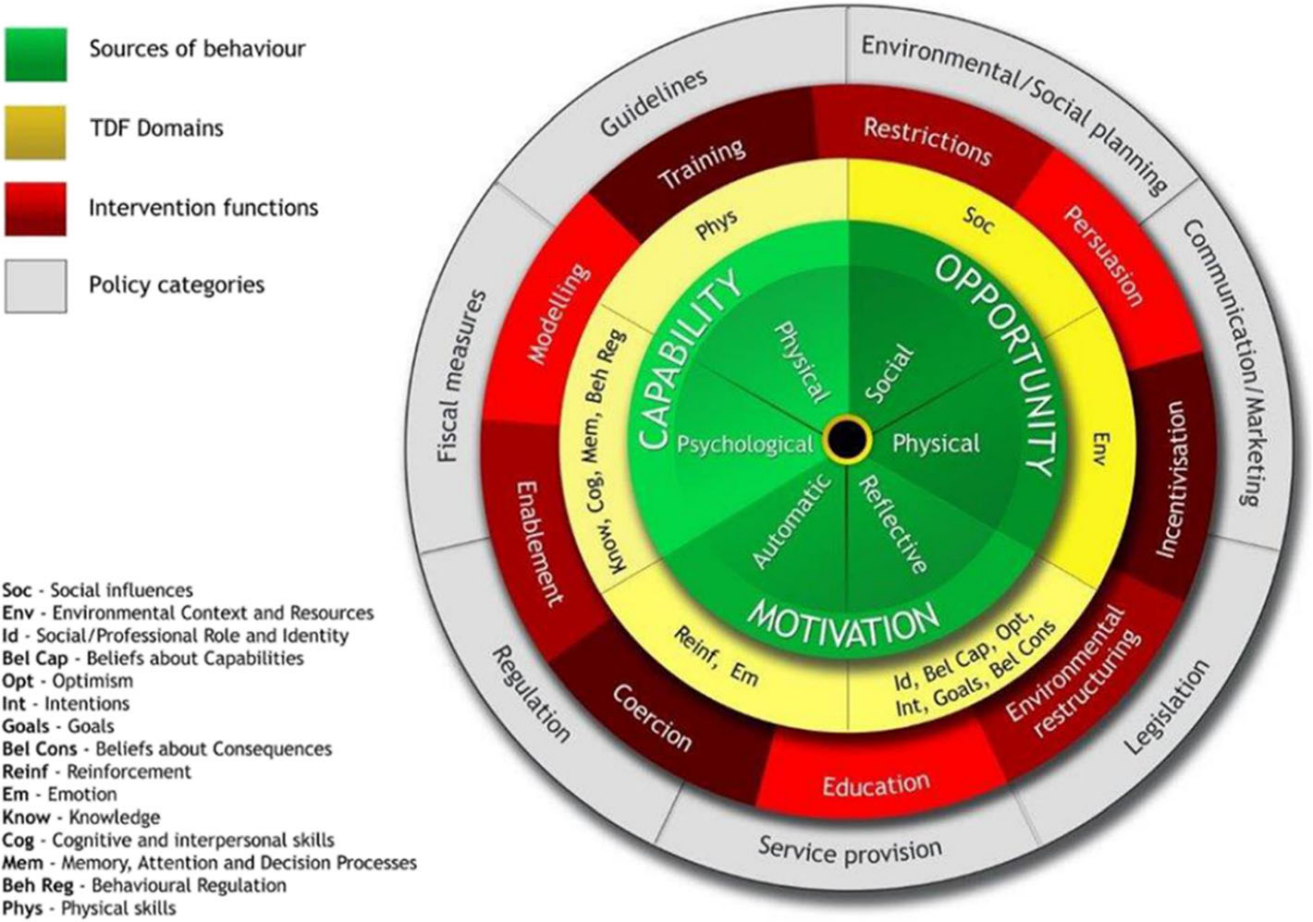
The Behaviour Change Wheel and Theoretical Domains Framework (TDF) domains. Reprinted with permission from Michie et al. [36, 37]

The Medical Research Council framework was used as a guide for the development and evaluation of the Activate intervention [42, 43]. The BCW was used to systematically develop the Activate intervention. We applied the BCW twice. Firstly, we applied the BCW to understand what hinders and facilitates patients in changing their level of physical activity. Secondly, we applied the BCW to analyse what behaviour change is needed in nurses to deliver the Activate intervention adequately. The BCW consists of three layers (see Fig. 3). In the first layer, we identified the source of the behaviour that could prove targets for the intervention (what needs to change) by conducting a COM-B analysis. To elaborate the behavioural analysis, we expanded on COM-B using the Theoretical Domains Framework (TDF) [44, 45]. The TDF is based on a synthesis of numerous overlapping theories of behaviour [45]. The 14 domains of the TDF can be mapped onto the capability, opportunity and motivation components of COM-B (see Fig. 3, Additional files 3 and 3). In the second layer, we used COM-B to generate a list of intervention function options. Intervention functions (e.g., education, persuasion) are broad categories of means by which an intervention can change behaviour. To determine which intervention functions to use, we applied the APEASE criteria (affordability, practicability, effectiveness and cost-effectiveness, acceptability, side effects/safety and equity). The third layer of the BCW identifies seven types of policy (e.g., guidelines, social planning, legislation) that can be used to deliver the intervention functions [36, 37]. This layer was not applicable to the present trial, because the intervention is not being implemented on a broad scale, but is being studied in a small number of practices. Finally, the intervention functions were linked to the BCTs described in BCTTv1, and we selected BCTs considering the APEASE criteria and available evidence of their effectiveness in the literature.

#### Applying the BCW to enhance physical activity in patients

We conducted a pragmatic literature review of qualitative studies to understand the behaviour of patients and to identify the perceived barriers to and facilitators of enhancing patients’ physical activity. The electronic databases MEDLINE and Embase were searched to retrieve publications of patients’ perceived barriers to and facilitators of increasing their level of physical activity. Also, relevant references in included papers were added. Possible relevant publications were assessed to extract perceived barriers and facilitators until saturation was achieved. Results of the review were mapped onto COM-B and TDF. In the second layer of the BCW, we selected intervention functions likely to be most effective in encouraging the target behaviour to occur (Additional file 3).

Subsequently, the intervention functions were directly linked to a selection of appropriate and effective BCTs, resulting in 17 BCTs (Table 1). This selection of BCTs is guided and peer-reviewed by experts in behaviour change. Each BCT is thoroughly designated for application in the Activate intervention following the definition of each BCT as described in the BCTTv1 taxonomy [38], resulting in four different consultations to enhance physical activity in the patients’ home environment. During this process, two focus groups were held with primary care nurses to apply the APEASE criteria and to validate the intervention for feasibility in practice.

**Table 1:** Selected behaviour change techniques for the Activate intervention and division of behaviour change techniques between consultations

Selected BCTs from BCTTv1 (examples of application of included BCTs)		BCTs divided between four consultations			
		1	2	3	4
1.	Goal setting (behaviour) (e.g., agree a personal daily activity goal)	x	x	x	x
2.	Problem solving (includes barrier identification and relapse prevention) (e.g., prompt to identify personal advantages and disadvantages of physical activity, focus on advantages and deal with disadvantages, discuss ways to prevent or deal with relapse)	x	x	x	x
3.	Goal setting (outcome) (e.g., agree on a personal health goal such as a decrease in patient’s blood pressure)	x			
4.	Action planning (e.g., prompt to plan specific activities at particular times during the week using the personal activity log)	x	x	x	x
5.	Review behavioural goal(s) (e.g., examine how the patient worked on the agreed goal, and consider re-setting, modifying or continuing with the agreed goal)		x	x	x
6.	Commitment (e.g., ask to affirm the agreed goal and action plan)		x	x	x
7.	Feedback on behaviour (e.g., give feedback using the personal activity log)	x	x	x	x
8.	Self-monitoring of behaviour (e.g., ask to wear the accelerometer and to fill in the personal activity log daily)	x	x	x	x
9.	Social support (unspecified) (e.g., encourage support from patient’s spouse or ‘buddy’)		x	x	x
10.	Social support (practical) (e.g., encourage practical help from patient’s spouse or ‘buddy’)		x	x	x
11.	Information about health consequences (e.g., inform about health benefits of physical activity)	x	x		
12.	Prompts/cues (e.g., advise to use the Post-Its and the pen with the study logo to remind of physical activity)		x	x	x
13.	Habit formation (e.g., prompt to rehearse and repeat the planned daily activities)			x	x
14.	Graded tasks (e.g., assist to increase the level of activity step by step by agreeing with achievable and challenging goals)		x	x	x
15.	Restructuring the physical environment (e.g., advice to repair the bike, buy good shoes or rain clothes)		x	x	x
16.	Restructuring the social environment (e.g., advise to go walking with a friend instead of drinking coffee)		x	x	x
17.	Focus on past success (e.g., encourage to think about occasions on which the patient succeeded in being physically active)	x	x	x	x

*Abbreviations*: *BCTTv1* Behaviour Change Technique Taxonomy v1, *BCT* Behaviour change technique

#### Applying the BCW to deliver the Activate intervention by primary care nurses

Subsequent to the development of the Activate intervention, we applied the BCW with nurses. In the first layer of the BCW, we explored qualitative literature by searching the electronic databases MEDLINE and Embase to retrieve publications on nurses’ perceived barriers and facilitators in delivering a behaviour change intervention and scrutinised reference lists of identified papers. Publications were assessed to extract perceived barriers and facilitators until saturation was achieved. In addition to the literature review, a focus group with primary care nurses was held to identify what nurses need to change to deliver the intervention. Prior to the focus group, nurses were asked to give their opinion, using a 4-point Likert scale (1 = totally disagree, 4 = totally agree), on the APEASE criteria regarding the 17 BCTs in daily practice and to reflect on their capability, opportunity and motivation to apply these BCTs. Results of the review were mapped onto COM-B and TDF. Results of the literature and focus group showed that all components of COM-B need to be targeted to adequately deliver the BCTs integrated into the Activate intervention (Additional file 4).

In the second layer of the BCW, we selected intervention functions (Additional file 4) and directly linked these to appropriate and effective BCTs (Table 2). This resulted in a selection of 21 BCTs. Each BCT is thoroughly designated for application in standardized training for nurses to equip them to deliver the intervention as intended. This process is peer-reviewed by experts and checked for face validity by four primary care nurses during a second focus group.

**Table 2:** Division of selected behaviour change techniques between the different components of the training of primary care nurses

Selected BCTs from BCTTv1 (examples of application of included BCTs)		BCTs divided between component training			
		Preparation	1-day training	Coaching sessions	Available resources
1.	Information about health consequences (e.g., inform about health benefits of physical activity using the background video, presentation during the 1-day training and the workbook)	x	x		x
2.	Information about social and environmental consequences (e.g., inform about the social and environmental consequences of increasing physical activity using the background video, a presentation during the 1-day training and the workbook)	x	x		x
3.	Prompts/cues (e.g., advise to use the Post-Its and the pen with the study logo, send monthly newsletter, have regular contact with nurses)		x		x
4.	Feedback on the behaviour (e.g., provide feedback on nurses’ performance during the role-plays and their audiotapes of the consultations)		x	x	
5.	Information about others’ approval (e.g., inform nurses about professionals’ and patients’ approval of their performance of their learned skills)		x	x	
6.	Credible source (e.g., all training components are developed and delivered by experts)	x	x	x	x
7.	Focus on past success (e.g., focus on what went well while [practising] delivering the consultations)		x	x	
8.	Verbal persuasion about capability (e.g., tell that nurses can successfully deliver the consultations, improve their skills by practising and feedback, and coach on self-doubts)		x	x	
9.	Reward (outcome) (e.g., nurses improve their coaching skills by participating in the study and the training is accredited)		x	x	
10.	Monitoring of behaviour by others without feedback (e.g., observe role-plays and listen to the audiotapes without feedback)		x	x	
11.	Monitoring outcome of behaviour by others without feedback (e.g., results from questionnaires, interviews with patients)		x		
12.	Instruction on how to perform the behaviour (e.g., train how to apply the BCTs using role-plays)	x	x	x	x
13.	Demonstration of the behaviour (e.g., demonstrate how to apply the BCTs using the instruction videos)		x	x	x
14.	Behavioural practice/rehearsal (e.g., prompt practice of applying the BCTs during the role-plays and the actual consultations)		x	x	x
15.	Habit formation (e.g., prompt repetition of applying the BCTs by including several eligible patients)		x	x	x
16.	Adding objects to the environment (e.g., provide a handbook with example sentences, Post-Its and a pen with the study logo, use patient’s daily activity log)		x	x	x
17.	Restructuring the physical environment (e.g., facilitate consultations to focus on solely physical activity, encourage use of the handbook with example sentences during the consultations, use patient’s daily activity log)		x	x	x
18.	Social support (unspecified) (e.g., encourage and coach regularly by mail and telephone, provide monthly newsletter)		x	x	
19.	Social support (practical) (e.g., provide nurses with all study materials and answer questions and remarks)		x	x	
20.	Problem solving (includes barrier identification and relapse prevention) (e.g., prompt to deal with lack of motivation and adherence to the study protocol)		x	x	x
21.	Self-monitoring of behaviour (e.g., prompt making audiotapes of consultations)		x	x	

*Abbreviations*: *BCTTv1* Behaviour Change Technique Taxonomy v1, *BCT* Behaviour change technique

#### The Activate intervention

The Activate intervention is developed for patients at risk for CVD. The intervention is standardised in four nurse-led consultations to enhance physical activity—in the first week and after 2, 6 and 12 weeks—and will take place in the patient’s own general practice. Although the BCTs are systematically applied in the intervention, the content of the intervention will be individualised to the patient’s unique circumstances, needs and preferences by adapting the BCTs during the consultations (e.g., goal setting, action planning, feedback). The duration of the first consultation is 30 minutes, and the subsequent consultations last 20 minutes. During the first consultation, patients will receive a workbook containing information about the study, useful websites and apps, tips and tricks, activity logs and action plans.

In the first consultation, the nurses will discuss the patient’s CVD risk profile, the consequences of a sedentary versus an active lifestyle, and self-assessment of their activity level in order to raise awareness to improve the patient’s level of physical activity. Together, the patient and nurse formulate an overall outcome goal and an exercise goal, considering physical activity in minutes per day. In order to raise awareness of self-monitoring and how to self-monitor, the patient is asked to self-monitor physical activity during the next 2 weeks by using an accelerometer and a paper activity log, which provide feedback on the patient’s level of goal attainment. Additionally, the patient is asked to identify facilitators to goal attainment.

During the second consultation, the nurse rehearses the information about the consequences of an active lifestyle, reviews the goal attainment using the activity log kept by the patient, and discusses the identified facilitators. If applicable, the physical activity goal is re-formulated. A specific action plan to attain the level of physical activity formulated as their goal is set up. The patient is supported in finding ways to use facilitators for physical activity, is asked to self-monitor activities during the next weeks in order to attain the set physical activity goal, and is asked to identify possible barriers to goal attainment.

In the third consultation, the nurse will give feedback on the reached level of goal attainment during the past weeks, using the log kept by the patient. If applicable, the activity goal and specific action plan are adjusted. Furthermore, the nurse will discuss how to prevent relapse into an inactive lifestyle (old habits) by discussing the barriers leading to relapse as well as how to build new habits to maintain the active lifestyle. The patient is asked to self-monitor activity level during the next weeks in order to attain the set physical activity goal and to identify possible barriers to and facilitators of goal attainment.

In the last consultation, the nurse will give feedback on the reached level of goal attainment during the past 6 weeks, using the results of the patient’s self-monitoring and activity log and the identified facilitators. Furthermore, the nurse will rehearse how to prevent relapse into old habits and the formation of new activity habits.

#### Training of primary care nurses

A comprehensive, standardised training for nurses is developed in collaboration with an educator and a health psychologist, in which the 21 BCTs are integrated (see Table 2). Prior to inclusion of patients, nurses allocated to the intervention arm will receive a 1-day, interactive, educational, face-to-face, accredited training in a small group outside the general practice, led by a health psychologist. To prepare themselves for the training, nurses will be asked to watch an instructional video of the study procedures and to watch a video of background information on the importance of physical activity in patients at risk for CVD provided by a physiotherapist.

At the start of the 1-day training, nurses will be asked to fill out a self-assessment and discuss their results in a group discussion. In this self-assessment, nurses will be asked to reflect on their capability, motivation and self-efficacy regarding each of the 17 BCTs integrated into the Activate intervention and their outcome expectancy for these BCTs on a 7-point Likert scale (1 = completely agree to 7 = completely disagree). This self-assessment is specifically developed for this study. The key focus of the 1-day training is learning how to address the BCTs in each consultation. Furthermore, the 1-day training entails an explanation of the intervention and its timeline, as well as information about the health consequences of physical activity. The training will contain a combination of didactic presentations, short videos on how to apply the BCTs in each of the consultations, small-group discussions and role-plays. Furthermore, nurses receive two individual coaching sessions by the health psychologist in which the trained skills in applying the BCTs are rehearsed and optimized. Prior to each coaching session, nurses will be asked to audiotape one of their consultations of the Activate study in their practice, which will be discussed during the coaching session.

The nurses will be provided with several resources, which they can use during their consultations. They will have a workbook for each patient in which charts (what to do when) and example sentences are given to help them deliver the BCTs effectively. In addition to the individual coaching sessions, nurses will be asked to watch the videos on how to apply the BCTs in the intervention to reinforce their skills and competencies in delivering the Activate intervention. The division of selected BCTs between the different components of the training is shown in Table 2.

### Feasibility and piloting

The training for nurses was pilot-tested in a small feasibility study. To evaluate whether the consultations were feasible in time and to ensure the intervention matched with the patient’s needs and could be incorporated into daily life, two primary care nurses completed the training, and one patient at risk for CVD completed the consultations. Their experiences, barriers, strengths, limitations and time investments were evaluated. The nurses indicated that the training adequately equipped them to deliver the intervention. They suggested minor adaptations, namely inclusion of patients across a broader age range (40–75 years), easier interpretation of level of adherence to the Dutch Norm for Healthy Exercise according to the SQUASH, and clear instructions on how to recruit and include patients. The time spent on the consultation was acceptable and in accordance with the protocol. The patient was satisfied with the intervention and suggested minor adaptations in the layout of the activity log.

### Care as usual

Patients in the control arm will receive care as usual, according to the national health care standards for patients at risk for CVD [16], during regular consultations with their nurse and will not receive additional consultations beyond standard of care to increase their physical activity level. Patients at risk for CVD have at least one consultation per year with their nurse; however, this frequency can be extended when considered necessary (e.g., in case of medication change). Patients with DM2 have at least four consultations with their nurse annually. In order to keep the nurses in the control arm motivated, the 1-day training will be offered to them at the end of the study.

### Data collection

To assess the characteristics of the nurses participating in the study, a short questionnaire will be sent to nurses. To assess whether patients are eligible for improving their level of physical activity, patients are asked to fill out a short self-assessment using the SQUASH prior to consenting to participate in the study. Nurses will interpret the completed SQUASH using clear guidelines to see if a patient fits the inclusion criteria regarding insufficient level of physical activity. After enrolment, patient data will be collected at baseline (T0), after 3 months (T1) and after 6 months (T2) by use of an accelerometer, questionnaires and chart review (see Fig. 2). At time point T0, the nurses will distribute the questionnaires and accelerometer after enrolment during their regular scheduled visit. At time points T1 and T2, the research team will distribute the questionnaires and accelerometers. To maximize retention of general practices, the research team will contact each general practice regularly, and nurses can easily contact the research team for remarks and questions. Nurses will receive a monthly newsletter to keep them updated on the number of recruited patients in the other attending general practices and to invite them to share their experiences with other nurses. To maximize retention of patients, the research team will contact patients by telephone or e-mail if no questionnaire and accelerometer are received within 3 weeks. If the research team is not able to contact the patients after several attempts, we will ask the attending nurse to contact the patient. Furthermore, patients are encouraged to contact the research team if they have remarks and questions.

#### Data management

Data collection as well as handling and storage of data and documents will be coordinated at the University Medical Center Utrecht. Entering of objective data collected from the accelerometers will automatically be uploaded from the accelerometer by two researchers on the research team. Entering of subjective collected data will be performed electronically by an independent data manager who is not aware of patient allocation.

### Outcome measures

#### Primary outcome

The primary outcome is physical activity objectively measured as the number of minutes of physical activity in the moderate to vigorous category. This will be assessed with a personal activity monitor (Pam AM300; Pam bv, Oosterbeek, The Netherlands) [46]. The Pam AM300 is a small, valid, and reliable triaxial accelerometer which can easily be worn on the hip. Additionally, patients will be asked to write down in a paper log the amount of minutes they have swum, cycled or done strength training, because the accelerometer cannot measure these activities accurately.

The number of minutes of physical activity in the moderate (3–6 metabolic equivalents [METs]) and vigorous (≥6 METs) categories at 6 month of follow-up will be considered as the primary outcome measure. Patients will be asked to wear the accelerometer during 7 consecutive days for 12 h daily at baseline (T0), at 3 months of follow-up (T1) and at 6 months of follow-up (T2). For a valid measurement, the accelerometer has to be worn for at least 4 weekdays and 1 weekend day for 8 h. After each data collection point, patients will be asked to send the accelerometer to the research team to upload the data from the accelerometer to a data file. The outcome is the average number of minutes of moderate to vigorous activity on all the valid days. With the Activate intervention, a mean difference in minutes of 20% of at least moderate level of physical activity from baseline is considered to be clinically relevant and reasonable to achieve within 3 months of intervention.

#### Secondary outcomes

The following are secondary outcomes of this trial:

1. Sedentary behaviour using the accelerometer to measure the number of minutes in the sedentary category (<1.8 METs)
2. Self-efficacy for physical activity using the Exercise Self-efficacy Scale [47–49]
3. Patient activation using the PAM-13 short form [50, 51]
4. Health status using the EQ-5D questionnaire [52]

Potential effect modifiers to investigate which patient characteristics modify change in physical activity level include age, depression measured using the Hospital Anxiety and Depression Scale [53, 54], body mass index (BMI), level of education, social support using the Multidimensional Scale of Perceived Social Support [55], patient-provider relationship using the Communication Assessment Tool [56], and baseline number of minutes of moderate to vigorous level of physical activity using the accelerometer.

#### Process evaluation

A mixed method process evaluation will be performed at the end of the study. To evaluate treatment fidelity [34, 57, 43], nurses allocated to the study arm will randomly audiotape one consultation from among the four consultations. The audiotapes will be coded using a coding list developed specifically for this study, consisting of the content of each of the four consultations and the Behaviour Change Counselling Index [58]. Additionally, nurses will be instructed to self-report the presence of the patient and the discussed content during a consultation, the time needed per consultation, and reasons for the patient’s dropout if applicable. To evaluate the nurses’ perceptions of their capability, motivation, self-efficacy and outcome expectancy of applying the BCTs integrated into the Activate intervention, nurses will be asked to complete the self-assessment at the start of the training, after the training, during the intervention period, and at the end of the study. At the end of the study, nurses in the intervention arm will be invited to a semi-structured interview to explore their experiences in delivering the intervention. Included topics are perceptions of the study procedures, barriers to and facilitators of implementation of the intervention, applying the BCTs, the training programme, self-efficacy, motivation of nurses and patients, perceived effect of the intervention, and their evaluation of the acceptability of the intervention for implementation in routine primary care. Furthermore, descriptive data will be collected to identify existing socio-demographic variation in who received the intervention and who dropped out of the intervention.

To explore patients’ experiences with the intervention, patients in the intervention group will be asked additional questions in the T1 questionnaire. To deepen our understanding of patients’ experiences, a sample of patients from the intervention group will be invited to a semi-structured interview at the end of the study. Included topics in the T1 questionnaires and interviews are perceptions of the outcome, capability, self-efficacy, motivation, intention, opportunity, barriers and facilitators, and satisfaction with the intervention.

#### Additional parameters

Patients’ socio-demographic characteristics, including sex, employment status, living alone/with others, years since diagnosis, smoking status, alcohol consumption and healthy food intake will be collected by using questionnaires at baseline. Blood pressure, cholesterol levels, glycated haemoglobin (DM2 only) and medication use will be extracted from the patients’ charts at baseline. Characteristics of nurses, including age, level of nursing education, number of years of experience working as a primary care nurse, number of years working in the field of CVRM, achievement of self-management training and geographical area of the general practice will also be collected at baseline.

### Statistical analysis

#### Effectiveness of the activate intervention

Data will be analysed primarily according to intention to treat and secondarily according to per-protocol principles. All patients with outcome data will be included in the intention-to-treat analysis, regardless of their adherence to the intervention. Patients in the intervention group will be included in the per-protocol analysis if they received a minimum of three consultations (75%), based on the registration forms obtained from the nurses. Patients from both groups were excluded from this analysis if they did not complete the T1 measurement. To examine the effect of the Activate intervention between the arms, a multilevel analysis will be performed (three levels: time, participant and general practice). The continuous outcome data will be quantified by mean, SD and 95% CI using linear mixed effects models. For the binominal outcome data, risk ratios and 95% CIs will be estimated using generalised estimating equations (GEEs) with a log-link function. In case of non-convergence of a model, ORs will be estimated using a logistic mixed effects model. All mixed effects models include a random intercept for changes over time and between practices. An interaction term will be added for time and study arm. Missing data will be handled according to the rules of the questionnaires of missing data. Missing outcome data will not be imputed, because multilevel analysis is a flexible method for dealing with missing outcome data [59]. Sensitivity analyses, such as an analysis of protocol deviations, definitions of outcomes, and outliers, will be performed to assess the robustness of the findings.

#### Potential effect modifiers

To examine which patient characteristics modify effectiveness of the intervention as reflected by increased physical activity level, pre-specified patient characteristics are selected, including age, BMI, level of education, social support, depression, patient-provider relationship and baseline number of minutes of physical activity. To identify potential effect modification, we will use GEEs for each of these patient characteristics separately. The independent variables in the models will be the patient characteristic, random intercept, interaction term for study arm and patient level. Effect modification will be considered significant if the interaction term shows a level of significance <0.05. All quantitative analyses will be performed using IBM SPSS Statistics for Windows version 21.0 software (IBM, Armonk, NY, USA).

#### Process evaluation

Quantitative data will be analysed using descriptive statistics. The audio recordings will be transcribed and independently analysed by two researchers using the coding list developed for this study. The interviews with nurses and patients to explore their experiences with the intervention will be audiotaped and transcribed. These transcriptions will be analysed and qualitatively described using thematic analysis as described by Braun and Clarke [60].

#### Sample size calculation

The present trial is powered to detect a mean difference between the intervention arm and control arm of 20% in number of minutes of at least a moderate level of physical activity. Based on the results of the It’s LiFe! trial [61], the mean level of physical activity in participants is 38 minutes (SD 18.1). The It’s LiFe! trial is a three-arm trial performed in primary care in the Netherlands with patients with chronic obstructive pulmonary disease and DM2 in which the researchers aimed to increase physical activity with a self-management support programme and the It’s LiFe! tool (a monitoring and feedback tool). This study revealed an increase in physical activity of 27%, but only in the counselling and It’s LiFe! tool group, objectively measured with the Pam AM300. We consider an increase in physical activity of 20% as reasonable to achieve. Taking a power of 80% and a significance level of 5% into account requires 89 patients per arm. Assuming an intra-cluster correlation of 0.05 and a cluster size of 8 patients per general practice requires 30 or 31 participating practices [59]. Allowing a patient dropout rate of 15%, we aim to recruit 279 patients in total and 9 or 10 patients per general practice.

#### Stopping rules

There are no formal stopping rules. If a patient decides to withdraw, the nurse will stop the intervention for that patient. Patients can withdraw from the study at any time.

#### Participant withdrawal

Patients can withdraw from the study without giving a reason. Nurses will monitor and report any adverse events to the research team and can advise discontinuation of the study in case of any adverse events. Patients who withdraw from the study before they have completed the T0 measurement will be replaced. Patients who withdraw after the T0 measurement will not be replaced.

## Discussion

Despite the growing evidence for their effectiveness, so far self-management interventions show small effects on health outcomes. The effectiveness of interventions is ambiguous, and the question who benefits most from these interventions is still unanswered. With the Activate study, we expect to shed light on the effectiveness of self-management interventions and explore which subgroup of patients benefits most. The effectiveness of this intervention and understanding which patients benefit from the intervention may lead to a broader application of this intervention in supporting patients to enhance behaviour change in other self-management components (e.g., dietary intake, alcohol use, medication adherence and smoking cessation).

The Activate intervention was comprehensively developed using the BCW and was applied to the behaviour of both patients and nurses. Because the role of a competent health care provider is essential in the delivery and fidelity of self-management interventions [33, 34, 62, 63], we aimed to equip nurses with training that supports them to increase their skills and competencies to adequately deliver the intervention.

This study has several strengths. We performed a detailed analysis of the behaviour of both patients and nurses. Subsequently, BCTs were selected and described, which will enhance reproducibility of the intervention. Furthermore, this cluster RCT is being conducted across several general practices in different urbanisation areas in the Netherlands with the patient’ s own primary care nurse, rather than trained researchers, delivering the intervention. This strengthens the generalisability and relevance of the findings from this trial for primary care. Another methodological strength is the use of the informed consent for postponed information procedure, which reduces selection bias by attrition or dropout of patients. In our study, the control group might be dissatisfied at not receiving the intervention, which would increase the risk of biased results. Changes in outcome may be affected only because of a demoralized and perhaps less motivated control group.

A methodological challenge is the objective measurement of the primary outcome by accelerometry. Because cycling, strength training and swimming are activities that cannot be measured with the accelerometer, we will ask patients to self-report engaging in these activities. However, these self-reported data will not be considered as primary outcomes. Furthermore, wearing the accelerometer might stimulate patients to be more active; however, these effects apply for patients in both the intervention and control arms. This might reduce the effect of the intervention. The use of the self-reported SQUASH for inclusion of patients will possibly lead to over-estimation of their level of physical activity, leading to fewer patients eligible for inclusion [64]. The training and consultations were pilot-tested in only two primary care nurses and one patient, which limits the insight into the barriers to and facilitators of performing the intervention.

### Trial status

The trial is in the data collection phase. Recruitment was started in March 2016 and was finished in January 2017.

## Additional files

Additional file 1: SPIRIT checklist. (PDF 130 kb)
Additional file 2: Informed consent form. (PDF 304 kb)
Additional file 3: Results of the behavioural analysis in patients to increase the level of physical activity using the BCW. (PDF 251 kb)
Additional file 4: Results of the behavioural analysis in primary care nurses to deliver the intervention using the BCW. (PDF 212 kb)

## Abbreviations

APEASE criteria: Affordability, practicability, effectiveness and cost-effectiveness, acceptability, side effects/safety and equity
BCT: Behaviour change technique
BCTTv1: Behaviour Change Technique Taxonomy v1
BCW: Behaviour Change Wheel
BMI: Body mass index
COM-B: Capacity, opportunity or motivation influencing behaviour
CONSORT: Consolidated Standards of Reporting Trials
CVD: Cardiovascular disease
CVRM: Cardiovascular risk management
DM2: Diabetes mellitus type 2
GEE: Generalised estimating equation
MET: Metabolic equivalent
Pam AM300: Personal activity monitor
PAM-13: Patient Activation Measure
SPIRIT: Standard Protocol Items: Recommendations for Interventional Trials
SQUASH: Short Questionnaire to Assess Health
TDF: Theoretical Domains Framework
Q: Questionnaire
C: Chart review
HLS-EU-Q: European Health Literacy Survey Questionnaire
MSPSS: Multidimensional Scale of Perceived Social Support
HADS: Hospital Anxiety and Depression Scale
CAT: Communication Assessment Tool
ESS: Exercise Self-efficacy Scale
HbA1c: Glycated haemoglobin
