# Supplementary material for: Unravelling effectiveness of a nurse-led behaviour change intervention to enhance physical activity in patients at risk for cardiovascular disease in primary care: study protocol for a cluster randomised controlled trial
Source: Trials. 2017 Feb 22;18:79. doi: 10.1186/s13063-017-1823-9 (PMC5322635; doi:10.1186/s13063-017-1823-9)
Supplement: Additional file 2: — Informed consent form. (PDF 304 kb) [file 13063_2017_1823_MOESM2_ESM.pdf]

Toestemmingsformulier In Actie studie

Hierbij verklaar ik dat ik:

- de informatiebrief voor de deelnemers van de In Actie studie (versie 28 januari 2016) heb gelezen. Ik begrijp de informatie.
- vrijwillig mee doe aan dit onderzoek. Ik weet dat ik op ieder moment kan beslissen om toch niet mee te doen. Daarvoor hoef ik geen reden te geven.
- aanvullende vragen kon stellen. Mijn vragen zijn genoeg beantwoord. Ik had genoeg tijd om te beslissen of ik meedoe.
- toestemming geef om de gegevens te gebruiken voor de doelen die in de informatiebrief staan.
- toestemming geef om de specialist(en) die mij behandelt te vertellen dat ik meedoe aan dit onderzoek.
- weet dat sommige mensen mijn gegevens kunnen zien. Deze mensen staan vermeld in de informatiebrief.
- weet dat mijn onderzoeksgegevens na het onderzoek nog 15 jaar bewaard worden en daarna worden vernietigd.
- weet dat mijn gegevens altijd vertrouwelijk zullen worden behandeld.

Ik wil na het onderzoek ☐ wel / ☐ geen informatie ontvangen over de resultaten van het onderzoek op groepsniveau.

Ik geef toestemming om in de toekomst opnieuw gevraagd te worden voor deelname aan nieuw onderzoek binnen hetzelfde onderzoeksgebied.

☐ ja / ☐ nee

Ik vind het goed om aan dit onderzoek mee te doen.

Naam : ……………………………………………………………….Dhr / Mw.

Straat / Huisnummer : ……………………………………………………………….................

Postcode / Woonplaats : ……………………………………………………………….................

Telefoonnummer : ……………………………………………………………….................

Geboortedatum : ……………………………………………………………….................

Emailadres (optioneel) : ……………………………………………………………………..

Handtekening: …………………………………………………....... Datum: _____ /_____ / _____

[Onderstaande tekst in te vullen door de POH]

Ik verklaar hierbij dat ik deze deelnemer heb geïnformeerd over het onderzoek. Als er tijdens het onderzoek informatie bekend wordt die de toestemming van de deelnemer zou kunnen beïnvloeden, dan breng ik hem/haar daarvan tijdig op de hoogte.

Naam : ……………………………………………………………………...............

Functie : ……………………………………………………………………...............

Plaats : ……………………………………………………………………...............

Handtekening: …………………………………………………....... Datum: _____ /_____ / _____
