# Supplementary material for: Unravelling effectiveness of a nurse-led behaviour change intervention to enhance physical activity in patients at risk for cardiovascular disease in primary care: study protocol for a cluster randomised controlled trial
Source: Trials. 2017 Feb 22;18:79. doi: 10.1186/s13063-017-1823-9 (PMC5322635; doi:10.1186/s13063-017-1823-9)
Supplement: Additional file 3: — Results of the behavioural analysis in patients to increase the level of physical activity using the BCW. (PDF 251 kb) [file 13063_2017_1823_MOESM3_ESM.pdf]

**COM-B**	**TDF**	**What needs to happen for patients to increase their level of physical activity***	**Intervention functions**	**BCTs**
Physical capability	Physical skills	- Have the skills and physical capability to walk (1-13)	Training	Feedback on behaviour, graded tasks, self-monitoring of behaviour
Psychological capability	Knowledge	- Have the knowledge and understanding of the influence of physical activity on the condition to reduce misperceptions and increase sense of urgency (8, 10, 14-20)	Education	Information about health consequences, feedback on behaviour
	Cognitive and interpersonal skills	- Have the skills to increase their level of physical activity (8, 10, 14-18)<br>- Have the skills to deal with conflicting or confusing recommendations (7, 10, 11)<br>- Have the skills to set goals, self-monitoring (e.g. wearing an accelerometer), and action planning (9, 19)	Training	Feedback on behaviour, habit formation, graded tasks, self-monitoring
	Memory, attention and decision processes	- Notice and remember to be physical active, make everyday decisions to exercise according to an action plan (3, 21)	Training, environmental restructuring, enablement	Feedback on behaviour, prompt and cues, habit formation
	Behavioural regulation	- Concordance to self-monitoring (e.g. wearing an accelerometer), and action planning (9, 19)<br>- Break well-established habits (6, 15)<br>- Have triggers to prompt (rewards, supervision, mail) (2, 17, 18, 22)	Education, training, enablement	Self-monitoring of behaviour, habit formation, action planning, prompts and cues
Physical opportunity	Environmental context and resources	- Have time to exercise (3, 6, 8, 9, 12, 13, 16, 18, 19)<br>- Have good weather or good shoes and clothes for all weather types (2-4, 8, 12, 18)<br>- Have alternatives to deal with bad weather, neighbour insecurities, transport problems (8, 18, 23)<br>- Improve easy access to affordable and stimulating facilities (at home or in their neighbourhood) tailored to the patients' needs and preferences (1, 2, 4, 18)<br>- Have a flexible routine allowing for an increase in walking (2, 9)<br>- Have the opportunity to be physical active during work (9)	Training, environmental restructuring, enablement	Problem solving, feedback on behaviour, restructuring the physical environment, restructuring the social environment, social support (practical), self-monitoring of behaviour
Social opportunity	Social influences	- Have positive support from family, friends, caregivers, fellow patients (e.g. have a buddy to exercise with or a buddy that supports exercise) (4, 6, 8, 9, 11, 13, 16, 19-21, 23-25)<br>- Have a collaborative relationship/ communication with caregiver (5, 8, 20, 23, 24)<br>- Have a competent caregiver (knowledge, clear guidance and stimulation, supervision, tailored advice, addressing importance of physical activity) (1-3, 11, 12, 25)<br>- Overcome culture and language barriers (16, 20)<br>- Have role models (25)	Environmental restructuring, modelling, enablement	Social support (unspecified), problem solving, restructuring the social environment

Reflective motivation	Professional/ social role and identity	Not applicable	Not applicable	Not applicable
	Beliefs about capabilities	- Overcome personal struggles (anxiety in unfamiliar surrounds, negative, or depressive emotions, body images) (5, 13, 18, 19, 21)<br>- Have insight in own behaviour (10)<br>- Have appropriate self and external monitoring (e.g. must be challenging, improve self-efficacy, provide feedback) (9)	Education, persuasion, enablement	Focus on past success, feedback on behaviour, self-monitoring of behaviour, problem solving, graded tasks, goal setting (behaviour), action planning, review behavioural goal(s)
	Optimism	- Cope with negative attitudes and experiences (10, 12, 26)<br>- Experience health benefits of increasing their level of physical activity (3, 4, 9, 13, 18)	Education, persuasion	Focus on past success
	Beliefs about consequences	- Believe that exercise is good, and has positive influences on their condition (9, 13)<br>- Believe that exercising helps to sleep well and lose weight (9)<br>- Experience health benefits of increasing their level of physical activity (3, 4, 9, 13, 18)	Education, persuasion, modelling	Information about health consequences, feedback on behaviour
	Intentions	- Motivated to change their physical activity level (2, 4, 6, 12, 15, 16, 18)<br>- Feel they want to take responsibility to be physically active (26)<br>- Feel the need/urgency to change their physical activity level (18)<br>- Perceive health as priority (1, 26)	Education, persuasion, incentivisation	Commitment, feedback on behaviour
	Goals	- Set achievable and personal goals (3)<br>- Action planning (9)<br>- Deal with conflicting goals (9)	Education, persuasion, incentivisation, enablement	Self-monitoring of behaviour, goal setting (behaviour), goal setting (outcome), action planning, review behavioural goal(s), problem solving, feedback on behaviour
Automatic motivation	Reinforcement	- Have positive prompts and cues in environment (16)<br>- Have routines and habits for daily exercising (6, 15)	Training, environmental restructuring	Prompts and cues, habit formation
	Emotion	- Enjoy being physically active (4, 6, 9, 13, 18, 25)	Incentivisation	Feedback on behaviour
*****Results of a review of qualitative studies<br>Abbreviations: BCTs: Behaviour Change Techniques; BCW: Behaviour Change Wheel; COM-B: Capability, Opportunity, Motivation, Behaviour; TDF: Theoretical Domains Framework				

References

1. Bethancourt HJ, Rosenberg DE, Beatty T, Arterburn DE. Barriers to and facilitators of physical activity program use among older adults. Clin Med Res [Internet]. 2014 Sep;12(1-2):10-20.

2. Casey D, De Civita M, Dasgupta K. Understanding physical activity facilitators and barriers during and following a supervised exercise programme in type 2 diabetes: A qualitative study. Diabet Med [Internet]. 2010 Jan;27(1):79-84.

3. Elley CR, Dean S, Kerse N. Physical activity promotion in general practice--patient attitudes. Aust Fam Physician [Internet]. 2007 Dec;36(12):1061-4.

4. Hartman JE, ten Hacken NH, Boezen HM, de Greef MH. Self-efficacy for physical activity and insight into its benefits are modifiable factors associated with physical activity in people with COPD: A mixed-methods study. J Physiother [Internet]. 2013 Jun;59(2):117-24.

5. Bayliss EA, Ellis JL, Steiner JF. Barriers to self-management and quality-of-life outcomes in seniors with multimorbidities. Ann Fam Med [Internet]. 2007 Sep-Oct;5(5):395-402.

6. Mancuso CA, Sayles W, Robbins L, Phillips EG, Ravenell K, Duffy C, Wenderoth S, Charlson ME. Barriers and facilitators to healthy physical activity in asthma patients. J Asthma [Internet]. 2006 Mar;43(2):137-43.

7. Rockwell JM, Riegel B. Predictors of self-care in persons with heart failure. Heart Lung [Internet]. 2001 Jan-Feb;30(1):18-25.

8. Albarran NB, Ballesteros MN, Morales GG, Ortega MI. Dietary behavior and type 2 diabetes care. Patient Educ Couns [Internet]. 2006 May;61(2):191-9.

9. Normansell R, Smith J, Victor C, Cook D, Kerry S, Iliffe S, Ussher M, Fox Rushby J, Whincup P, Harris T. Numbers are not the whole story: A qualitative exploration of barriers and facilitators to increased physical activity in a primary care based walking intervention. BMC Public Health [Internet]. 2014;14:1272-.

10. Jansink R, Braspenning J, van der Weijden T, Elwyn G, Grol R. Primary care nurses struggle with lifestyle counseling in diabetes care: A qualitative analysis. BMC Fam Pract [Internet]. 2010;11:41-.

11. Bratzke L, Muehrer R, Kehl K, Lee K, Ward E, Kwekkeboom K. Self-management priority setting and decision-making in adults with multimorbidity: A narrative review of literature. Int J Nurs Stud [Internet]. 2015;52(3):744-55.

12. Booth ML, Bauman A, Owen N, Gore CJ. Physical activity preferences, preferred sources of assistance, and perceived barriers to increased activity among physically inactive australians. Prev Med [Internet]. 1997 Jan-Feb;26(1):131-7.

13. De Bourdeaudhuij I, Sallis J. Relative contribution of psychosocial variables to the explanation of physical activity in three population-based adult samples. Prev Med [Internet]. 2002 Feb;34(2):279-88.

14. Jansink R, Braspenning J, Keizer E, van der Weijden T, Elwyn G, Grol R. Misperception of patients with type 2 diabetes about diet and physical activity, and its effects on readiness to change. J Diabetes [Internet]. 2012 Dec;4(4):417-23.

15. Booth AO, Lowis C, Dean M, Hunter SJ, McKinley MC. Diet and physical activity in the self-management of type 2 diabetes: Barriers and facilitators identified by patients and health professionals. Prim Health Care Res Dev [Internet]. 2013 Jul;14(3):293-306.

16. Fitzgerald N, Spaccarotella K.
Barriers to a healthy lifestyle: From individuals to public Policy—An ecological perspective Journal of Extension. 2009;47(1)

17. Schutzer KA, Graves BS. Barriers and motivations to exercise in older adults. Prev Med [Internet]. 2004 Nov;39(5):1056-61.

18. Lascar N, Kennedy A, Hancock B, Jenkins D, Andrews RC, Greenfield S, Narendran P. Attitudes and barriers to exercise in adults with type 1 diabetes (T1DM) and how best to address them: A qualitative study. PLoS One [Internet]. 2014 Sep 19;9(9):e108019.

19. Simmons D, Lillis S, Swan J, Haar J. Discordance in perceptions of barriers to diabetes care between patients and primary care and secondary care. Diabetes Care [Internet]. 2007;30(3):490-5.

20. Nam S, Chesla C, Stotts N, Kroon L, Janson S. Barriers to diabetes management: Patient and provider factors. Diabetes Res Clin Pract [Internet]. 2011;93(1):1-9.

21. Rockwell JM, Riegel B. Predictors of self-care in persons with heart failure. Heart Lung [Internet]. 2001 Jan-Feb;30(1):18-25.

22. Cable TA, Meland E, Soberg T, Slagsvold S. Lessons from the oslo study diet and anti-smoking trial: A qualitative study of long-term behaviour change. Scand J Public Health [Internet]. 1999 Sep;27(3):206-12.

23. Johnston S, Irving H, Mill K, Rowan M, Liddy C. The patient's voice: An exploratory study of the impact of a group self-management support program. BMC Fam Pract [Internet]. 2012;13:65-.

24. Nagelkerk J, Reick K, Meengs L. Perceived barriers and effective strategies to diabetes self-management. J Adv Nurs [Internet]. 2006 Apr;54(2):151-8.

25. Allender S, Cowburn G, Foster C. Understanding participation in sport and physical activity among children and adults: A review of qualitative studies. Health Educ Res [Internet]. 2006 Dec;21(6):826-35.

26. Coventry P, Fisher L, Kenning C, Bee P, Bower P. Capacity, responsibility, and motivation: A critical qualitative evaluation of patient and practitioner views about barriers to self-management in people with multimorbidity. BMC Health Serv Res [Internet]. 2014;14:536-.
