# Supplementary material for: Unravelling effectiveness of a nurse-led behaviour change intervention to enhance physical activity in patients at risk for cardiovascular disease in primary care: study protocol for a cluster randomised controlled trial
Source: Trials. 2017 Feb 22;18:79. doi: 10.1186/s13063-017-1823-9 (PMC5322635; doi:10.1186/s13063-017-1823-9)
Supplement: Additional file 4: — Results of the behavioural analysis in primary care nurses to deliver the intervention using the BCW. (PDF 212 kb) [file 13063_2017_1823_MOESM4_ESM.pdf]

COM-B	TDF	What needs to happen for primary care nurses to adequately deliver the intervention	Intervention functions	BCTs
Physical capability	Physical skills	NA	NA	NA
Psychological capability	Knowledge	- Have the knowledge about physical activity (1)<br>- Have the knowledge to perform the BCTs #<br>- Have the knowledge to activate a patient and what to do when patients complain of physical pain #<br>- Have the knowledge to educate patients on a didactic way (1, 2)	Education	Information about health consequences, information about social and environmental consequences, feedback on behaviour
	Cognitive and interpersonal skills	- Have the skills to flexible and sensible tailor interventions and device alternative strategies and encourage patients to overcome barriers (1, 3)<br>- Have the skills to deliver BCTs # (1, 2)<br>- Have the skills to change their communication style (3, 4)<br>- Have the skills to educate patients on a didactic way (1, 2)<br>- Have the skills to flexible and sensible tailor interventions and device alternative strategies and encourage patients to overcome barriers (3)<br>- Have the skills to deal with patients' excuses, lack of motivation or have physical complaints, which makes it difficult for the nurse # (1)<br>- Need education and exercise to know how to develop a concrete and structured action plan # (1, 3)<br>- Let the patient participate; not filling in for the patient # (2, 3)<br>- Develop transferable skills for use with other patients (5)	Training	Feedback on behaviour, habit formation, demonstration of the behaviour, instruction on how to perform the behaviour, self-monitoring of behaviour, behavioural practice/ rehearsal, reward
	Memory, attention and decision processes	NA	NA	NA
	Behavioural regulation	NA	NA	NA
Physical opportunity	Environmental context and resources	- Have time to support patients during consultations # (1, 4, 5)<br>- Have the tools to perform the BCTs (to self-monitor, overview of physical activity options in the area, information brochures websites, apps, clear protocol, etc) # (2, 3, 5)	Training, environmental restructuring, enablement	Restructuring the physical environment, adding objects to the environment, social support (practical), instruction on how to perform the behaviour, prompts and cues, problem solving
Social opportunity	Social influences	- Have support to participate from general practice (e.g. time and education opportunities) # (5)	Environmental restructuring, enablement	Social support (unspecified)

		- Self-management is encouraged throughout the entire general practice # (2)<br>- Have the autonomy in planning their own work (2)		
Reflective motivation	Professional/ social role and identity	- Understand that activating a patient is part of the nurses' role and not necessarily of a physiotherapist or health facilitator# (1, 3)<br>- Belief that patients are suitable candidates for behaviour change (2)	Modelling, education, persuasion	Information about others' approval, feedback on behaviour
	Beliefs about capabilities	- Have tools to deliver the intervention that are easily and readily fit into daily practice (2, 3)<br>- Have comprehensive and effective learning methods (role-plays, follow-up sessions, credible source, clear instructions, role-play scenarios, written and verbal feedback) (3, 5)<br>- Feel confident that they can do it even the patient is not motivated #	Education, persuasion, modeling, enablement	Credible source, verbal persuasion about capability, demonstration of the behaviour, focus on past success, feedback on behaviour, self-monitoring of behaviour
	Optimism	NA	NA	NA
	Beliefs about consequences	- Expect that supporting patients in changing their behaviour is effective (1, 5)	Education, persuasion, modelling	Information about health consequences, information about social and environmental consequences, feedback on behaviour, focus on past success
	Intentions	- Have a positive attitude toward disease management and seriousness of the disease (4)<br>- Have a positive attitude toward collaborative care (4)<br>- Want to use new tools in practice (2)<br>- Feel that they are making a difference#<br>- Motivated to support patients in changing their behaviour (1, 5)<br>- Feel the need to change their routine practice (2)	Education, persuasion, incentivisation	Feedback on behaviour, monitoring of behaviour by others without feedback, monitoring outcome of behaviour by others without feedback
	Goals	NA	NA	NA
Automatic motivation	Reinforcement	NA	NA	NA
	Emotion	NA	NA	NA

Results from focus group with primary care nurses
Abbreviations: BCW: Behaviour Change Wheel; COM-B: Capability, Opportunity, Motivation, Behaviour; TDF: Theoretical Domains Framework; BCT: Behaviour Change Technique; NA: not applicable

References

1. Jansink R, Braspenning J, van der Weijden T, Elwyn G, Grol R. Primary care nurses struggle with lifestyle counseling in diabetes care: A qualitative analysis. BMC Fam Pract [Internet]. 2010;11:41-56.

2. Kennedy A, Rogers A, Bowen R, Lee V, Blakeman T, Gardner C, Morris R, Protheroe J, Chew-Graham C. Implementing, embedding and integrating self-management support tools for people with long-term conditions in primary care nursing: A qualitative study. Int J Nurs Stud [Internet]. 2014 Aug;51(8):1103-13.

3. Beighton C, Victor C, Normansell R, Cook D, Kerry S, Iliffe S, Ussher M, Whincup P, Fox Rushby J, Woodcock A, Harris T. "It's not just about walking.....it's the practice nurse that makes it work": A qualitative exploration of the views of practice nurses delivering complex physical activity interventions in primary care. BMC Public Health [Internet]. 2015;15:1236-.

4. Nam S, Chesla C, Stotts N, Kroon L, Janson S. Barriers to diabetes management: Patient and provider factors. Diabetes Res Clin Pract [Internet]. 2011;93(1):1-9.

5. Taylor C, Shaw R, Dale J, French D. Enhancing delivery of health behaviour change interventions in primary care: A meta-synthesis of views and experiences of primary care nurses. Patient Educ Couns [Internet]. 2011;85(2):315-22.
